# Barriers to and facilitators of coalface primary care reform in England: a qualitative study

**DOI:** 10.3399/BJGPO.2025.0065

**Published:** 2026-01-28

**Authors:** Claire Jackson AM, Caroline Nicholson, Jenny Job, Jon Sussex, Steven Morris

**Affiliations:** 1 General Practice Clinical Unit, University of Queensland, Queensland, Australia; 2 Centre for Health System Reform and Integration, University of Queensland, Queensland, Australia; 3 RAND Europe, Eastbrook, UK; 4 Department of Public Health and Primary Care, University of Cambridge, Cambridge, UK

**Keywords:** health policy, general practice, workforce

## Abstract

**Background:**

Since the Declaration of Alma-Ata in 1978, global health policy has prioritised primary and integrated care reform to better manage chronic illness, improve health access, and prevent disease. Yet internationally, primary care, like the health systems it struggles to support, is currently far from strengthened and is increasingly challenged by chronic underfunding, lack of recognition, and a diminishing and demoralised workforce.

**Aim:**

To better understand the policy barriers responsible for the current status from the perspective of general practice in England.

**Design & setting:**

Key informant interviews between August and October 2024 with 12 general practice policy or practice leaders identified from publicly available position statements, publications, or innovative programmes in UK primary care reform over the past decade.

**Method:**

A qualitative deductive approach using thematic analysis to analyse informant data to understand historical barriers and explore facilitators for future reform.

**Results:**

The analysis resulted in eight main themes: 1) dynamics of power and autonomy; 2) underinvestment in primary care; 3) aligning policy and implementation; 4) navigating complexity and change; 5) building trust through relationships and leadership; 6) the revolving door of policy and leadership; 7) valuing the workforce: a key to morale and retention; and 8) strategic communication and media engagement.

**Conclusion:**

Better-targeted funding reform, more effective systems integration building on general practice and community service strengths, and better valuing the complex role of the primary care sector as central to a high-functioning health system were seen as key to the future. Participants also called for more effective policy input from those skilled in the delivery of care and the capacity for earned autonomy and flexibility to deliver care relevant to individual community need. Action to address these opportunities is pressing, as finalisation of the 10 Year Health Plan and more immediate NHS restructure rapidly gathers momentum.

## How this fits in

Previous international literature has identified common barriers to engagement of primary care by policymakers, including trust and relationships, issues of autonomy, challenging change proposals, and lack of capacity. There is, however, limited recent investigation from a UK perspective. This study reinforced these themes within an English context, while also highlighting additional themes of underinvestment, lack of alignment between policy and implementation, workforce turnover, and communication exchange as highly relevant. Such themes deserve further exploration within the development of the NHS 10 Year Health Plan and in ongoing health services research internationally.

## Introduction

Since the Declaration of Alma-Ata in 1978,^1^ global health policy has prioritised primary and integrated care reform to better manage chronic illness, improve health access, and prevent disease.^2^ Yet internationally, primary care is currently far from strengthened and is increasingly challenged by chronic underfunding, lack of recognition, and a diminishing and demoralised workforce.^3,4^ Despite the global calls and commitments for effective health service redesign, tangible change at the patient care interface remains limited.^5,6^ While international literature has identified common barriers to engagement of primary care by policymakers including lack of trust, poor relationships between primary care and decision makers, physician autonomy, challenging change proposals, and lack of capacity, there is limited recent investigation of reform determinants from an English perspective.^7–9^

Since 2010, primary care in the NHS in England has undergone major structural and organisational reforms, shaped by strategic policy frameworks such as the 2014 *Five Year Forward View* ^10^ and the 2025 mandate to NHS England.^11^ These reforms aimed to modernise service delivery, improve integration, and ensure sustainability in a publicly funded universal healthcare system.

The *Five Year Forward View* was a pivotal document that called for a *‘radical upgrade in prevention and public health’* and introduced new models of care.^10^ It laid the groundwork for primary care networks (PCNs), which were formally launched in 2019.^12^ PCNs group general practices into collaborative units serving 30 000–50 000 people, enabling shared staffing, extended services, and a stronger focus on population health.

Building on this, integrated care systems (ICSs) were formalised in 2022, replacing clinical commissioning groups (CCGs). ICSs brought together NHS providers, local authorities, and voluntary organisations to plan and deliver services across regions, aiming to shift the system from competition to collaboration.

The 2025 mandate to NHS England^11^ reinforced these changes by prioritising access, digital transformation, and neighbourhood-level care. It supported reforms such as expanding the roles of allied health professionals, modernising GP contracts, and enhancing data sharing to reduce administrative burdens.

From an international perspective, these reforms resemble accountable care organisations in the US or local health networks in Canada. However, they are implemented within the context of the NHS’s publicly funded universal healthcare system. In general, the reforms aimed to preserve equity while enhancing efficiency, responsiveness, and sustainability in the face of rising demand and workforce pressures.

Despite these reforms, Lord Darzi’s 2024 review of the NHS depicted systemic service failure, decreasing patient satisfaction, poor system integration, and dwindling primary care capacity. He highlighted the disparity between hospital and general practice resourcing and poor GP access as key challenges.^13^

Has flawed primary care policy design and implementation contributed to this outcome and could grass-roots input inform a more effective approach? This study took a qualitative approach to better understand the policy barriers responsible for the current status from the perspective of general practice in England and used thematic analysis to deduce facilitators and solutions for a more successful future reform approach.

## Method

The primary aim of this study was to understand the major barriers to and facilitators of the implementation of effective primary care policy reform in England at the GP/patient level. The study focus was from the perspective of primary care thought leaders actively involved in implementation reform over the past decade.

### Design and setting

This study followed a qualitative deductive approach, using key informant data gathered from semi-structured interviews.

#### Participants

Study participants were general practice policy or practice leaders identified from significant publicly available position statements, publications, or innovative programmes in primary care reform over the past decade. They were also recruited via PCN innovation programmes, the Royal College of General Practitioners (RCGP) policy leadership positions, and via snowballing. Three were past-chairs or senior office bearers of the RCGP, three led PCN innovation initiatives, one was a Harkness Fellow, and two were internationally published on primary care reform. Seven were active in general practice within NHS England. Eleven of the interviewees were from England but a Scottish-based leader was also included to explore alignment with broader UK themes. Invitees were approached via email and all 12 accepted the invitation to participate. Further participant demographics are provided in Table 1.

**Table 1.** Participant characteristics

Participant	Sex	GP	Active practice	Published	Location	Age, years	Years in practice	Years active in PC policy
1	M	Y	N	Y	North-West England	>60	>20	>20
2	F	Y	Y	Y	South-East England	40–60	15	10
3	M	Y	N	Y	East Anglia	>60	>20	>20
4	M	Y	N	Y	London	>60	>20	>20
5	F	N	N	Y	West Midlands	>60	N/A	>20
6	M	Y	Y	N	North-West England	40–60	>20	10
7	F	Y	Y	Y	London	>60	>20	>20
8	F	Y	Y	N	North-West England	40–60	15	10
9	F	Y	Y	N	East Anglia	40–60	<10	5
10	M	Y	Y	Y	South-East England	40–60	>20	15
11	M	Y	Y	N	South-West England	40–60	15	5
12	F	Y	Y	N	Scotland	40–60	>20	15

F = female. M = male. N = no. N/A = not applicable. PC = primary care. Y = yes.

#### Sample size

As an alternative to data saturation, Malterud *et al*’s process was used to inform the sample size.^14^ Information power from the interviews was considered strong as the research questions were specific, the interview guide was informed by theory, interviewees were leaders in primary care, and the interviewer was experienced. Once the purpose and goals of the analysis were achieved, the sample size was determined to be met.

### Interview guide development

**Figure 1.**
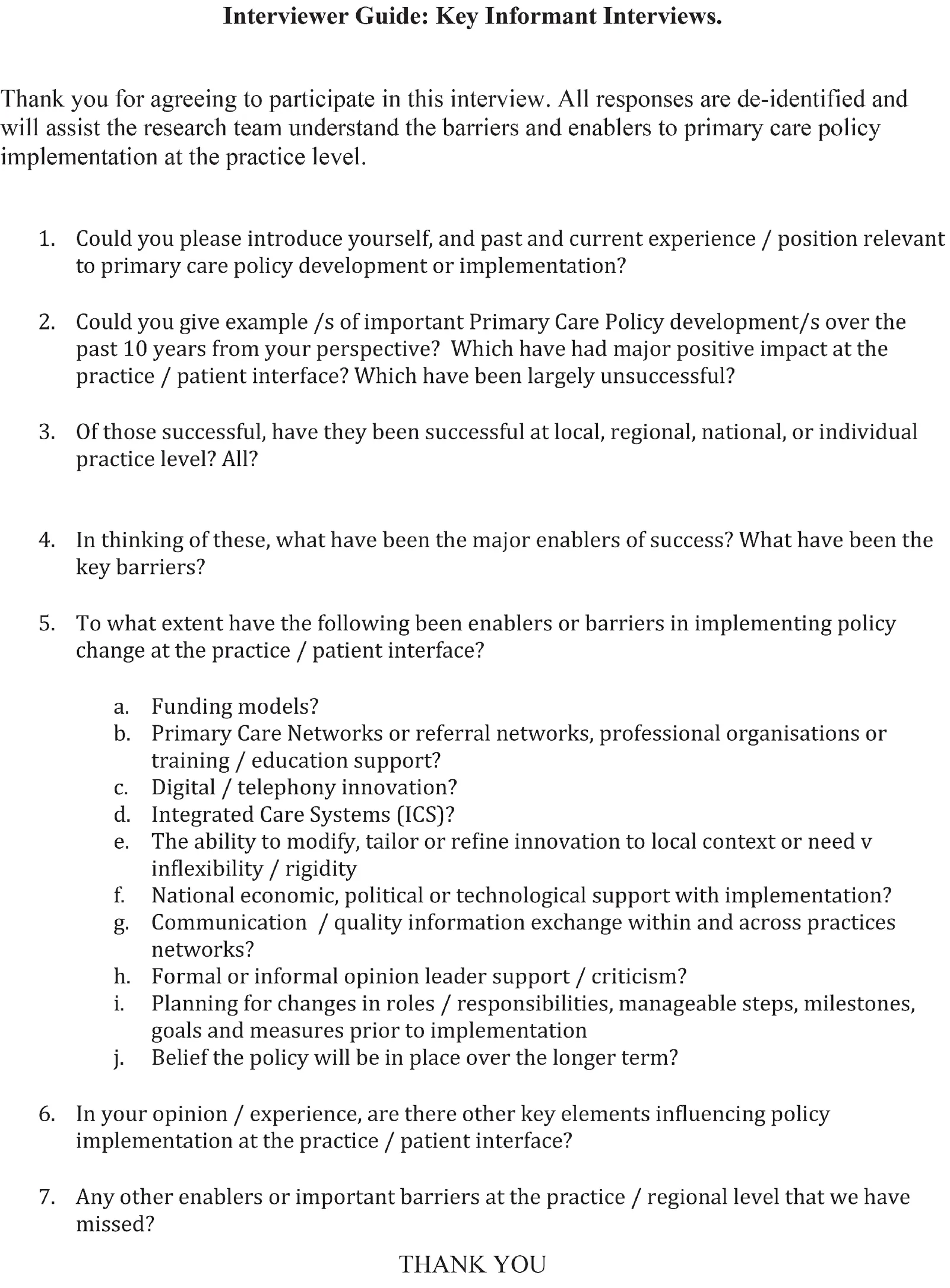
Interview topic guide

The interview guide was informed by the Consolidated Framework for Implementation Research (CFIR).^15^ CFIR offers a practical structure to guide knowledge of the complex, multi-level facets of implementation contexts, enabling the identification of factors that influence implementation across multiple settings. Two researchers (CJ and CN) experienced in primary care reform implementation reviewed the constructs of the five CFIR domains (innovation, outer and inner setting, characteristics of individuals involved, and process), choosing those most relevant across the primary care health setting to inform the interview format.^16^ Interview questions (Figure 1) were developed using the Interview Guide Tool available via the CFIR website (https://cfirguide.org). The interview guide also included an introductory question regarding perceptions of effective UK reform policy implementation, and concluded with open-ended questions regarding additional factors.

### Interviews

All interviews were conducted via Zoom by a single interviewer (CJ), a GP academic and policy advocate, experienced in semi-structured interview technique. Interviews lasted between 45 and 70 minutes and were undertaken between August and October 2024. Notes were recorded during and after interviews to identify barriers to and facilitators for implementing primary care reform. Interview questions were used to frame the interview, but interviewees were free to take the focus they wished in discussing important policy facilitators or barriers. Interviews were audio-recorded, transcribed verbatim using Microsoft Word, cross checked against the recording, and anonymised.

### Analysis

A reflexive, deductive thematic content analysis was used to identify patterns of meaning across the data material. A recursive analysis process was guided by the six phases outlined by Braun and Clarke:^17^ familiarisation with the data, generating codes, generating initial themes, reviewing themes, defining and naming the themes, and writing a contextualised narrative. Two members of the research team (CJ and CN) with experience in qualitative research analysed the transcript data independently and resolved disparities through discussion. Following the initial independent coding, the research team met to revise and clarify the coding structure, with the final categories derived through a consensus approach. Results were reported according to consolidated criteria for reporting qualitative research (COREQ).^18^

## Results

The analysis resulted in eight main themes, consistent across all 11 interviews with English participants and with no noted variation due to participant sex, age, or geography. No additional themes were noted in responses by the single non-English (Scottish) participant. Only content from English (NHS) participants is included in the following analysis.

### Dynamics of power and autonomy

Participants universally believed that while what they saw as underinvestment and funding inequity seemed the more obvious reform barriers (Theme 2), the chief perpetuating element lay in the lack of systems-level power to enable effective primary care resourcing and service delivery:

*‘Government has consistently said we are continuing to put more ... resource into hospitals and less into general practice and we're going to change that. And then they've done the reverse.’* (Participant [P]10)

*‘The set up of almost everything in the NHS gives power to acute trusts* [the organisations supplying secondary and tertiary hospital services] *over everything else.’* (P2)

*‘You have no power or authority ... ICBs* [integrated care boards, regional organisations responsible for commissioning health care services] *are meant to be the new drivers of change ... but ... they are not much about primary care.’* (P7)

*‘Until that lack of political will to make inroads into primary/secondary care funding split happens, I don’t really see ... change.’* (P6)

Participants felt that the most effective reform had occurred when funding allowed general practices to focus on their own population needs and use innovation and flexibility to deliver effective reform. They believed that well-resourced, population-focused autonomy within appropriate policy guidelines would be a major facilitator of effective reform:

*‘When groups of practices have been given some budget or resource to improve or develop primary care in a way that works best for their local population ... ’* (P5)

*‘GPs using the budgetary or policy opportunities that come past ... being able to use initiatives to develop super partnerships and federations and alternative contracts ... had flexibility to do some different things ... ’* (P5)

*‘What is missing is earned autonomy at the local level ... where a practice or primary care network can show it is walking the walk, then they should have more of a say in how the resources get utilised within their communities ... ’* (P6)

*‘How to maintain innovation of GPs to meet the needs of their populations is a tension … the need to control has actually snuffed out the need to innovate at an individual practice level ... ’* (P3)

### Underinvestment in primary care

Responders universally nominated ongoing primary care underinvestment and funding inequity (the byproduct of this power/autonomy imbalance) as the source for much of the current reform impasse:

*‘But actually, what’s happening is that the Department of Health and Social Care* [the Government ministry responsible for the NHS in England] *are actually spending less on primary care. So, you know, there’s not enough GPs, the rate of GPs is increasing at a much lower rate than our NHS consultants.’* (P8)

*‘The proportion of NHS funding on primary and community services is now at a record low.’* (P2)

*‘Providing resources is really important … the resources going into general practice as a percentage of total NHS spend is dropping dramatically … every time more new money goes into the NHS, it preferentially goes into the … acute sector.’* (P4)

The lack of integration with secondary care was also seen as problematic:

*‘And then we think about the development of integrated neighbourhood teams where with multiple different employers and multiple different contracts and multiple different funding streams and multiple different targets. How are you supposed to bring those together to address local projects? And the biggest issue about funding streams is this primary care/secondary care divide.’* (P6)

*‘We're not really being given enough resources to deliver the service that is needed. And I think the system hasn't taken into account the huge increase in GP workload and the issues caused by secondary care.’* (P8)

*‘Every time there’s an initiative about management in the NHS it tends to ignore primary care. It’s thinking about hospitals again.’* (P5)

### Aligning policy and implementation

A mismatch between policy development/design and implementation was another powerful theme:

*‘But I think there is a mismatch, isn't there, between the sort of people who are leading on health policy and people who are working at the coal face.’* (P8)

*’ICBs should be ... funding general practice … investing in general practice … strategically thinking about the role of general practice, particularly around population health for their communities … What they've actually ended up doing is just becoming a new tier of performance management.’* (P4)

*‘There does need to be a national overarching policy … but then I think it absolutely should be allowed to be interpreted locally ... ’* (P8)

*‘I would understand ... if you were to risk offering so much flexibility around policy, then it may not be put into practice in the way that you want ... why that variation can cause trouble … if there was more bridging between the national and the local, I think, it would be really helpful rather than landing on your desk with nothing in between.’* (P9)

All responders raised the establishment of PCNs as a significant general practice reform whose implementation has been challenging. Participants reported that PCNs’ impact had been limited as they were generally too small, significantly under-resourced, and often unable to translate important reform due to insufficient power:

*‘The lack of resources has been a problem for PCNs ... they don’t really hold much power in the system in the way CCGs did.’* (P4)

*‘PCNs have come to a certain level ... but they need additional investment and empowerment to bring those integrated neighbourhood teams on.’* (P6)

*‘When I speak to PCNs ... most say we spend most of our time running around chasing small aliquots of money writing bids ... so it’s a really poor way to create sustainable change.’* (P1)

*‘One of the problems of primary care networks, in my view, is they're too small because they're not big enough to provide organisation support ... and that’s a huge gap.’* (P10)

*‘But it’s probably stalled at the moment ... there’s much, much more those PCNs could do … there’s been a lot of top-downism telling PCNs what they should focus on — more of a political directive than actually what communities would prioritise if they could.’* (P6)

Some questioned whether more direct funding to practices might unlock greater innovation:

*‘The funding comes through them* [PCNs] *and they basically divvy up staff, and, as it were, allocate them to practices. So, there is a question whether … an alternative would be that the practice gets the money and decides who* [which role relevant to the practice population] *to employ ... there’s a great deal of controversy about how well this works.’* (P10)

### Navigating complexity and change

A perceived lack of understanding of the complexity of the sector by policymakers and funders was another consistent theme, as was a lack of training for change management support. Responders believed that policy development that was better grounded in real-world practice and service reality would be more successful:

*‘The complexity of GP funding is a real issue ... because we’re independent businesses ... fixed outgoings, staff ... our incomes are so variable year on year ... and it’s not sustainable.’* (P6)

*‘Some patients are very complex … the patient comes in and wants to chat about their agenda and the computer is flashing up the 10 tasks I need to complete that are attached to funding … ’* (P6)

*‘General practice policy is really complicated and historically maybe been slightly overlooked.’* (P2)

*‘There’s always the tension between what government wants and what general practice wants. Practices operate as a small business so always complicated for a Ministry of Health.’* (P5)

*‘Having people who understand the complexity of general practice at high level, who turn the whole thing upside down and have primary care driving the system not secondary care* [change]*.’* (P7)

The pace and poor management of the change process was another consistent theme:

*‘Think there’s change fatigue out there. There really is.’* (P8)

*‘Support with implementation? Lip service, that’s all we get … ’* (P7)

*‘Inevitably practices are missing out on funding and policy change … because their managers are overwhelmed managing the day to day of a business … ’* (P3)

*‘Because it feels like sometimes at national level they want the change, but they aren't prepared to put in the capacity to support doing it.’* (P5)

### Building trust through relationships and leadership

Responders identified a lack of policymaker trust in general practice and its business model and poor relationship building between general practice and policymakers as other significant underlying impediments to reform:

*‘A perception from government ... if you put money into general practice you can’t be sure what’s going to come out … frankly I think it’s been a fundamental barrier for underinvestment ... ’* (P2)

*‘We gave PCNs a tiny bit of money that we didn’t performance manage and that trust enabled them to do things.’* (P1)

*‘But we've* [PCNs] *been trusted ... to use it in the way that we thought was best ... we've had to ... share some key deliverables but these were put in place collaboratively, not as part of some directive.’* (P8)

*‘And I think it’s been really helpful because we know our communities, we know lots of the issues. So, therefore, it makes sense for us getting those resources and being supported to work with them … ’* (P8)

Working relationships over time and effective leadership were other sub-themes:

*‘The success we’ve had has been where human beings have been in place for a long time and those relationships.’* (P6)

*‘When there’s been really good leadership in our departments of health or social care, good civil servants who really understand primary care.’* (P5)

*‘When you do manage to find a way of engaging local clinicians, finding the best leaders, the people who are really inspired and take people with them, then that works exceptionally well ... ’* (P4)

### The revolving door of policy and leadership

Responders also identified the constant turnover in policy direction and in the responsible personnel as an ongoing barrier to reform implementation:

*‘There’s been so much churn — not just at the commissioning level from clinical commissioning groups to ICBs with huge loss of institutional memory and local knowledge ... but also within primary care ... GP Federations working together … then the PCNs came along and smacked them out of the ballpark.’* (P2)

*‘There is a lot of concern that the commissioning support for general practice has been lost in the transition from CCGs to ICBs and therefore that we're simultaneously faced with the need to really support general practice, and an absence of the support mechanisms for that.’* (P2)

*‘Where it hasn’t worked well is with the acute services ... they are very big and robust and their management teams are constantly changing ... so those relationships don’t ever really get strengthened.’* (P6)

### Valuing the workforce: a key to morale and retention

The morale and workload of the GP workforce were also seen as major barriers to reform implementation:

*‘Workload will come into all of this, you know. If we had more time, more capacity, more resource. We could all do so much better.’* (P8)

*‘I think one of the barriers is workload, so it’s very hard to sit back and think how you'd redevelop the system when you've still got 10 patients sitting in your waiting room.’* (P10)

*‘GPs just don’t have the headspace ... focus now is on survival.’* (P5)

*‘That demand has increased, number of GPs functioning has decreased, and they're really struggling.’* (P8)

Low morale was seen to be a particular risk:

*‘They* [GPs] *just feel really ignored and unloved. I know there’s issues of pay here but I don’t think it’s just about that ... it’s linked with the* [COVID-19] *pandemic — they were all saints yet afterwards there’s a lot of public dissatisfaction with problems of access. Practitioners remain exhausted.’* (P5)

*‘The biggest financial risk of any healthcare system is the morale of the staff and that’s never been lower.’* (P1)

*‘So, over a period of three years we went from being the only people out facing this deadly virus, taking it home to our families and all the rest of it, to being the reason that healthcare service is falling apart ... I'm 53 now, and a lot of my colleagues are just saying, jack it, this is not worth it ... We go to work and we get a kicking every day.’* (P11)

### Strategic communication and media engagement

The patchy packaging and delivery of key reform information was seen to be a major barrier, as was the perception that public figures promise services that are not possible. This placed practices at odds with patient expectations, created relational damage, and increased workload for the practice frontline:

*‘Politicians have a way of saying things that really heighten the temperature, like every patient should be able to see their GP within two days ... causes conflict between patients and their practice.’* (P6)

*‘All the GPs up and down the country have felt battered by the previous government who have used general practice as a bit of a scapegoat for years of failing to adequately resource the NHS.’* (P6)

*‘There’s a new policy initiative come out ... and that’s great and I can see how good it is. But it’s taken so long to get the clarity only this week — the day before that funding was due to be released.’* (P9)

*‘So we haven’t educated the public about the specialist role of the GP ... there’s some of the “I want a quick fix immediately.”’* (P3)

*‘With all due respect, it’s very useful to know the background, how something came about ... But I just need the bottom line.’* (P11)

Both mainstream and social media were often seen to be highly critical of reform initiatives, which created negativity and confusion:

*‘Often just anything that comes out is instantly portrayed as very negative in some way. And so then, that will affect people’s perception of* [reform] *schemes like that.’* (P9)

*‘I do think ... the negative voices just tend to get more traction on social media.’* (P9)

*‘And certainly when I talk to my colleagues and peers there, you know, there are lots of these GP social media type groups where everyone moans. I have to say I had to come out of it because I couldn't handle it.’* (P11)

## Discussion

### Summary

The NHS finds itself at a critical point in its mission to provide universal, high-quality care to UK residents. General practice, while central to a modern-day NHS, has benefited little from recent reforms and is seen to be chronically overstretched, demoralised, and under-resourced.^4^ Despite government policy aimed at increasing the number of UK GPs, there are 1500 fewer fully qualified GPs in the NHS today than 7 years ago,^19^ and there is growing consumer discontent with the lack of access. The resulting reduced engagement and capacity in the sector has had a direct flow-on to an already stretched acute care sector, especially emergency departments and complex care hospitalisation. This comes at a time when major reorganisations of the health service involving the abolition of NHS England and cuts to ICBs will have a considerable impact on primary care.

Our study participants identified failures in primary and integrated care policy implementations over the past decade contributing to this position, and proposed solutions possible within the existing NHS framework.

The eight themes identified were consistent across all interviewees. Although only the 11 English responders were included in the analysis for this study, the Scottish participant raised the same elements. While chronic primary care under-resourcing was a resounding theme throughout this study, so was the underlying cause as seen by all participants: the long-standing power imbalance between primary care and the much more influential and resourced hospital sector. ICSs and ICBs were seen as currently ineffective in delivering the service integration and funding reform required, compounded by a lack of understanding of the complexity of general practice service delivery by those designing policy at all levels.

The workforce turnover in those developing and implementing policy (politicians, NHS executives, commissioners, and hospital managers) was also seen to consistently derail most reform processes. The frequency of management staff turnover was seen to cause confusion, delay, or reversal, waste both fiscal and human resources, and hinder the all-important relationship building, trust, and unity of purpose required to deliver effective change.

The GP workforce was seen as increasingly demoralised and devalued, with a high level of burnout. Participants noted this as an immediate threat to the essential NHS reforms required and one for urgent action.

Messaging to those attempting to implement coalface reform was seen as often ineffective due to poor targeting and an audience with a heavy patient care load. Both mainstream and social media were seen as highly negative in their treatment of reform initiatives, colouring the perceptions of both the public and political audience.

The final two interview questions invited participants to nominate any additional reform facilitators or barriers that had not been covered. The impact of the pandemic was raised by two interviewees and the importance of continuity of care by one interviewee; however, other topical areas such as the shift to online working, establishment of mega-practices, and relative decline of the partnership model were not identified.

In addition to increasing general practice’s share of the NHS budget and immediate attention to growth in the general practice workforce, much better policy implementation synergy was seen to be at the heart of effective reform. This needed to better link those with current coalface experience with those designing, resourcing, messaging, and delivering policy change. ICBs needed to better understand and engage with capability in the GP sector and appropriately fund integrated care initiatives that deliver quality outcomes for patients locally and reduce acute demand. PCNs were seen as modestly successful but needed to be much larger and more powerful within the wider system to perform. A key facilitator at the local interface was seen to be a focus on *‘trust within guardrails’* (P6), better balancing general practice accountability and performance management, and resourcing innovative care options for those demonstrating the ability and partnerships to deliver them.

### Strengths and limitations

This study added to the limited publications on the topic, reinforcing previously reported themes and identifying new ones. Eleven of 12 study interviewees were GPs, thus policy reform impacts were seen from their perspective and inherent bias. The perspectives of other stakeholders (GP partners, the broader primary care workforce, commissioners, and patients) are important for a holistic understanding of system-level challenges and should form the basis for further research.

### Comparison with existing literature

While our study has identified similar themes to those cited in the international literature regarding barriers to engagement of primary care by policymakers,^7^ it has also identified new themes relevant in the current context.

Established areas such as lack of trust, poor relationships between primary care and decision makers, physician autonomy, challenging change proposals, and lack of capacity were all reinforced as key themes within participant interviews, mirroring previously reported elements.^7^ However, our study participants also highlighted the additional themes of underinvestment, lack of alignment between policy and implementation, workforce turnover, and communication exchange as highly relevant.

### Implications for research and practice

Such themes deserve further exploration within the development of the NHS 10 Year Health Plan, as the active engagement of general practice will be essential to reform success. They also merit investigation in ongoing health services research outside the UK, as the topic is one of international concern.

This study identified key facilitators of more effective reform from the perspective of primary care leaders involved in policy development over the past decade.

Better targeted funding allocation, more effective systems integration, building on general practice and community service strengths, and valuing the centrality of the complex role of the primary care sector were seen as key to the future. Participants also called for greater policy input from those skilled in the delivery of care, and earned autonomy and flexibility to deliver care relevant to individual community need. Action to address these opportunities is pressing, as finalisation of the 10 Year Health Plan and a more immediate NHS restructure gathers momentum.
